# Estimation of Some Phytochemical Compounds and Antioxidant Properties of Leaves from Different Mulberry Varieties Grown in Syria

**DOI:** 10.1155/2023/9929276

**Published:** 2023-07-29

**Authors:** Tahani Alidee, Maysam Jales, Nouraldin Daher Hjaij, Alaa Almouna

**Affiliations:** ^1^Department of Food Technology, General Commission of Scientific Agricultural Research (GCSAR), Damascus, Syria; ^2^Department of Entomology, Research Protection Plant of Administration, General Commission of Scientific Agricultural Research (GCSAR), Damascus, Syria

## Abstract

The study was conducted to compare the chemical composition and bioactive compounds of leaves from different mulberry species grown in Syria (*Morus alba*, *Morus nigra*, *Morus rubra*, and *Morus nigra* sp). The leaves were collected in July 2022 and their proximate composition (moisture, ash, lipids, and protein), total polyphenol content (TPC), ascorbic acid, and antioxidant activity, as measured by ferric reducing antioxidant power (FRAP), were investigated. The results showed that the moisture, ash, and fat content ranged from 61.84 to 71.17%, 12.87 to 17.61%, and 3.82 to 9.68% dry weight (DW), respectively. The phenolic concentration of leaves from four different mulberry cultivars varied significantly, from 164.9 (*Morus alba*) to 461.5 gallic acid equivalents mg/100 g DW (*M. nigra*). The antioxidant activity of mulberry leaves ranged from 122 to 166.3 meq ascorbic acid/100 g DW and was arranged as follows: *M. rubra* > *M. nigra* sp > *M. nigra* > *M. alba*. According to the findings, mulberry leaves could be used to create new food supplements, functional foods, and medical applications.

## 1. Introduction

The market for natural food products is one area of the health food sector that is currently expanding quickly. This is because the plant contains a wide variety of bioactive compounds with antioxidant properties which may help in the prevention of many chronic diseases [1].

The mulberry tree is a member of the *Moraceae* family and is cultivated in many Asian countries [2]. Mulberry is a woody plant that grows in a variety of climatic, topographical, and soil conditions and can be found all over the world [3]. Today, it is grown in Southwest Asia and Southern Europe, and it is noted as one of the most important fruits in the Mediterranean [4]. The most well-known species of the *Morus* genus are white mulberry (*Morus alba*), black mulberry (*Morus nigra*), and red mulberry (*Morus rubra*) [5]. Mulberry genotypes are very diverse in Syria, as they were sometimes obtained from seeds or cuttings in the past. This process has resulted in many landraces being adapted to various conditions and uses across the country. There are many local traditional accessions in Syria, but no named cultivars. Syrians distinguish and refer to the mulberry by using local names that are often based on the color of the fruit, such as “Abyad” (for a white mulberry), “Ahmar Baladi” (for a red mulberry), “Shami,” and “al-Masry” (black mulberry).

Different uses have been created for this plant's various morphological parts (leaves, fruits, roots, and stems). Most of the information is focused on mulberry fruits and leaves, which have medicinal qualities and are frequently consumed as part of a typical diet [6].

Mulberry leaves are important to the sericulture sector because they are the only food source for the silkworm (*Bombyx mori*). The leaves are also used in dairy animal feed because they increase milk production [7]. Mulberry leaves have been used in many countries as a medicine, nutritious beverage, and functional food [8]. For more than 3,000 years, mulberry leaves have been used in traditional Chinese medicine. They have long been used to treat many diseases such as colds and diabetes [9]. Modern research has shown that mulberry leaves contain polysaccharides, flavonoids, alkaloids, volatile oils, and other active components [10]. The active ingredients in mulberry leaves can be used to treat many diseases, including hypertension, diabetes, hyperlipidemia, Alzheimer's disease, and immunomodulation [11]. The Ministry of Public Health of China has currently approved mulberry leaves as pharmaceutical/food resources [12]. Gupta et al. [7] confirmed that mulberry leaves have a higher protein content than other green leafy vegetables. Therefore, mulberry leaves can be used as an excellent source of nutrients and fight malnutrition in many underdeveloped areas of the world [13]. The mulberry leaf's nutritional and functional content was significantly affected by cultivar, harvesting time, and degree of maturity. Also, the functional compounds of mulberry leaves vary between varieties, according to a previous study [14]. Therefore, it is important to choose the right mulberry leaves through activity studies based on variety. Iqbal et al. [15] evaluated the chemical composition and bioactive compounds in the leaves of three mulberry cultivars and found that there were significant variations in some nutrients and bioactive compounds between the various mulberry cultivars.

To date, no research has been carried out on the chemical composition and antioxidant activity of mulberry leaves cultivated in Syria. Therefore, the purpose of this study was to determine the proximate composition, total phenols, and antioxidant activity of leaves from four Syrian mulberry cultivars.

## 2. Materials and Methods

### 2.1. Chemicals and Reagents

In this study, the following reagents were used: gallic acid standard (GAE), Folin–Ciocalteu's phenol reagent, sodium carbonate, 2,6-dichloroindophenol, D-glucose, L(+)-Ascorbic acid, and 1,1-diphenyl-2-picrylhydrazyl (DPPH) were obtained from Sigma Aldrich (German). Phenol (99.5%, Riedel-de Haën, Germany), sulphuric acid (95–98%, Panreac Quimica SA, Spain), oxalic acid (99%, Panreac Quimica SA, Spain), methanol (99.5%, Panreac Quimica SA, Spain), potassium ferricyanide (98%, Avonchem, United Kingdom), trichloroacetic acid (99.5%, Sigma-Aldrich, Germany), Hexan (≥97%, Riedel-de Haën, Germany), and ferric chloride (98%, SRL, India).

### 2.2. Leaf Samples Collection

The fresh leaves (young) of four mulberry species were collected in July 2022. The leaves of Al-Abyad *(Morus alba)*, Al-Masry (*Morus nigra* sp), and Ahmar Baladi (*Morus rubra*) were harvested from mulberry fields in Damascus city (Basatin Abu Jarash, 33°31′51.6″N 36°17′31.6″E), while the Al-Shami (*Morus nigra*) leaves were collected from Khan Arnabah (Al Qunaitra Governorate, southern Syria), Syria. The largest glossy leaves from the tops of the branches were collected from several randomly selected trees of the same species after the fruits had reached full maturity. The sampling was performed only once to avoid the different environmental and climatic conditions that affect the natural phytochemical compounds.

After collection, the leaves are removed from the stalk, washed with water, and shade dried to constant weight. The samples were kept in a dry place, away from light, until they were analyzed.

### 2.3. Proximate Composition

The proximate composition of leaf samples (moisture, ash, lipids, and protein) was analyzed using the AOAC [16] method. The moisture was determined by drying the samples in an oven drier at 105 ± 2°C until a constant weight was obtained. The ash content was determined using the ashing method, which involved heating the sample in a furnace. A Soxhlet extractor was used to extract lipids from powdered samples. The nitrogen content was estimated using the Kjeldahl apparatus, and the protein content was calculated as *N* × 6.25.

### 2.4. Total Soluble Carbohydrate (%)

The total soluble carbohydrate in the leaf samples was determined according to the method of Xiao et al. [17] with some modifications. The mulberry leaf powder (1 g) was dissolved in distilled water at a ratio of 1 : 10 g/mL and extracted in a water bath for 20 min at 60°C. The aqueous solution (1 mL) was mixed with 1 mL of the phenol solution (5%), followed by 5 mL of concentrated sulphuric acid. After 10 min, the reaction was thoroughly mixed with a vortex and placed in a water bath at 30°C for 20 min. The absorbance was measured at 490 nm with a UV-VIS Spectrophotometer (PG Instruments T80+ UV/VIS Spectrophotometer, UK). The standard curve of D-glucose ranging from 10 to 100 *μ*g/mL was prepared.

### 2.5. Determination of Ascorbic Acid Content

Ascorbic acid was determined according to the method of Dinesh et al. [18] with some modifications. A fine-dried powder (1 g) was extracted with 20 ml of oxalic acid (0.4%) and stirred at room temperature for 20 min. The mixture was filtered through Whatman No. 4 filter paper. The sample extract was titrated against a solution of 2,6-dichloroindophenol (0.02%) until the pink color of the solution was sustained for 10 s. The dye solution was standardized with 10 mL of ascorbic acid (0.2 mg/mL). The ascorbic acid content of the samples was expressed as mg/100 g of DW leaves.

### 2.6. Preparation of Phenolic Extract

One Gram of the dried leaves was extracted with 15 ml methanol (70%) for 1 h at ambient temperature under continuous stirring in a magnetic stirrer. After filtration, the extracts were stored at −20°C until further analysis.

### 2.7. Determination of Total Polyphenol Content (TPC)

The TPC was determined using the Folin–Ciocalteu calorimetric method described by Kostic et al. [19]. The phenolic extract (1 mL) was combined with 0.5 mL Folin–Ciocalteu reagent and 2 ml sodium carbonate (20%). After 10 min of incubation at room temperature, the absorbance was measured at 765 nm. The total phenolic of the extracts was calculated as mg of gallic acid equivalents/100 g dry leaves (mg GAE/100 g DW).

### 2.8. Ferric Reducing Antioxidant Power (FRAP) Assay

The Mekonnen and Desta [20] method was used to determine the FRAP assay. One ml of the phenolic extract was mixed with 2.5 mL of a phosphate buffer solution (pH = 6.6) and 2.5 mL of potassium ferricyanide (1%). After the mixture was incubated at 50°C for 20 min, 2.5 ml of trichloroacetic acid (10%) was added. Then, 2.5 mL of the mixture was taken, and 2.5 mL of water and 0.5 ml of ferric chloride (0.1%) were added. Finally, the absorbance was measured at 700 nm against a blank. The L-ascorbic acid (0.5–2.5 *μ*g/mL) was used to prepare the calibration curve. The results were expressed as mg of ascorbic acid/100 g dry sample (mg AA/100 g DW).

## 3. Statistical Analysis

The results were expressed as mean ± standard deviation (SD). The statistical analysis was carried out using GenStat software 12 and a one-way analysis of variance (ANOVA) followed by a Fisher's least significant difference (LSD) test to determine the significant difference between the treatments at the 5% level.

## 4. Results and Discussion

### 4.1. Proximate Composition

Table 1 illustrates the mean compositional values for the leaves of four different mulberry species.

The moisture content varied between 61.84% in *M. nigra* and 71.17% in *M. rubra*. According to the data presented by several authors, the average moisture of mulberry leaves ranges between 60% and 75% [21, 22]. Low moisture content may contribute to leaf roughness [15].

The moisture content in dried leaf powder ranged from 2.66% to 6.33%. Low moisture content in mulberry leaves enhances the extraction of bioactive compounds and helps protect the product from microbial attack and spoilage [23]. The total mineral content is reflected in the total ash content, and a high ash content indicates that plant material has high inorganic nutrients [24]. The ash content of dried mulberry leaves varied significantly in all experimental types except between *M. alba* and *M. rubra*. The ash content in mulberry leaves was lowest in *M. nigra.* Srivastava et al. [13] have achieved similar results.

The protein content of mulberry leaves can be used to determine the leaf's quality. The trend of protein concentration in leaves from all the cultivars studied was as follows: *M. nigra* > *M. alba* > *M. nigra* sp > *M. rubra*, with significant differences between them (*p* < 0.05). Sanchez-Salcedo et al. [21] found slightly lower levels (13.4% to 19.4%) in leaves of *M. alba* and *M. nigra*, while Srivastava et al. [13] found higher values in leaves of six genotypes of mulberry in India. Yu et al. [8] illustrated that mulberry leaves contain high-quality proteins which are used to fortify food in the subcontinent.

Estimating lipids is one of the most important aspects of any material's nutritional evaluation [25]. In the four analyzed mulberry leaves, the fat content ranged from 3.82% dw (*M. alba*) to 9.68% DW (*M. nigra* sp). The ranges for fat in mulberry leaves given by specific literature are 4.24–6.75% [15] and 2.09–6% [13].

Mulberry leaves' total soluble carbohydrate was analyzed (Table 1). The finding showed that the investigated mulberry extracts had a total soluble carbohydrate content that ranged from 1.77 to 3.57% DW. *M. alba* and *M. rubra* had the highest soluble carbohydrate, while *Morus nigra* had the lowest concentration. Our findings are lower than that of the previously reported data [13].

### 4.2. Total Phenolic Compounds (TPC)

The concentration of TPC in the different types of mulberry leaves studied is presented in Table 2. The concentration of phenols in the four mulberry cultivars' leaf extracts ranged from 164.9 to 461.5 mg/100 g DW. Furthermore, the data indicate that *Morus nigra* had a significantly higher TPC than other cultivars. This study's findings agreed with a report on mulberry leaves from Pakistan and Italy [6, 15], which also indicated that black mulberry had higher total phenolic compound values than white mulberry. Polumackanycz et al. [6] reported that the TPC for 19 varieties of mulberry leaf ranged from 11.17 to 31.14 mg GAE/g. While Yu et al. [8] found that TPC levels in mature leaves of different mulberry varieties grown in China ranged from 27.63 to 37.36 mg/100 g DW. This variation could be explained by the difference between extraction procedures and analytical methods used in each study. Furthermore, the phenolic compounds in leaves change in response to environmental factors such as drought, temperature changes, pollution, UV light, and pathogen attacks, among others [26].

The ascorbic acid content in leaves ranged from 1.66 (*M. nigra* sp) to 3.84 mg/100 g DW (*M. nigra*) (Table 2). This result is consistent with the findings of Flaczyk et al. [27]. Srivastava et al. [13] found higher levels of ascorbic acid in the leaves of some mulberry (*M. alba*) genotypes, ranging from 100 to 200 mg/100 g DW. Iqbal et al. [15] observed that the highest concentration of ascorbic acid was found in the leaves of *M. alba* followed by *M. nigra* while the minimum was found for *M. rubra*. The variations in nutritional compound levels between leaves of mulberry varieties or within the same variety can be attributed to pedoclimatic factors (soil type, sun exposure, and precipitation), genetic factors (variety), and agricultural variables (organic farming, tree fruit production, the state of maturation, growing area, fertilization, and irrigation) [28].

### 4.3. Antioxidant Activity

**Figure 1:**
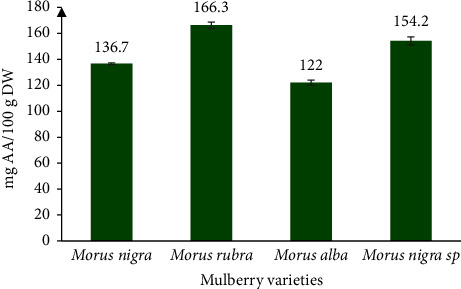
Antioxidant activity of mulberry leaves.

The antioxidant activity of food products is an important indicator of their health benefits [29]. The FRAP assay was used to determine the total antioxidant activity of mulberry leaves. This method depends on the extract's capacity to reduce the ferric (Fe^3+^) to the ferrous (Fe^2+^) state. The results of antioxidant activity in Figure 1 showed statistically significant differences between the mulberry varieties (*p*  <  0.05) and *Morus rubra* which had the highest reducing capacity (166.3 mg ascorbic acid equivalent/100 g DW). According to Cartea et al. [30], the chemical structures of compounds, particularly the number and positions of aromatic and hydroxyl groups, have a significant impact on the antioxidant activity of phenolics. The antioxidant activity is also correlated with the degree of hydroxylation. A previous study showed that the FRAP values of white mulberry aqueous extracts were higher than those of black mulberry. Furthermore, no significant differences in antioxidant activity were observed between the white and black mulberry alcoholic extracts [6]. According to Wang et al. [2], there are many factors that can affect the antioxidant activities of plant samples, including extraction method, extraction solvent, and assay system.

### 4.4. Correlation between Different Experimental Parameters

Table 3 shows the correlation coefficient between the experimental parameters investigated for the different mulberry leaf varieties. A positive correlation (*r* = 0.61) was found between TPC and antioxidant capacity. This suggests the important role of phenolic compounds as antioxidant constituents in the mulberry extracts. Many studies observed a good correlation between TPC and antioxidant activity [6, 8]. According to Yu et al. [8], the polyphenolic compounds in mulberry leaves are chlorogenic acid, rutin, isoquercitrin, and astragalin, and they are primarily potential antioxidants. Protein correlated negatively with all other parameters observed except ascorbic acid (*r* = 0.40). Our results agree with those reported by [8], who found a significantly negative correlation between TPC and crude protein. The high correlation between fat and FRAP (*r* = 0.87) is possibly due to the presence of *α*-linolenic acid, linoleic acid, palmitic acid, and oleic acid in mulberry leaves [31]. The FRAP assay was found to be negatively correlated with ascorbic acid (*r* = −0.35). Iqbal et al. [15] revealed a weak correlation between ascorbic acid and antioxidant activity in mulberry leaves.

## 5. Conclusions

For the first time, some chemical parameters, phenolic compounds, and antioxidant activity of Syrian mulberry leaves were investigated in this study. According to the findings, *Morus* leaves are a good dietary source of nutritional and bioactive compounds. This study suggests that the leaves of Al- Shami mulberry had the highest protein and phenol content (*p* < 0.05), whereas the leaves of Al-Ahmar Baladi (*Morus rubra*) had significantly higher antioxidant activity compared to the other mulberry species. Our findings indicated that Syrian mulberry leaves might be a promising source of natural antioxidants for the food, pharmaceutical, and cosmetic industries due to the presence of phenolic compounds. Further research is needed to isolate and identify the antioxidant components in mulberry leaves.

## Figures and Tables

**Table 1:** Mulberry leaves' chemical compositions.

Chemical compositions	*Morus nigra*	*Morus nigra* sp	*Morus rubra*	*Morus alba*
Moisture (FW%)	65.61 ± 0.42^b^	61.84 ± 0.41^a^	71.17 ± 0.15^d^	68 ± 0.63^c^
Moisture (DW%)	5.05 ± 0.04^b^	2.66 ± 0.23^a^	3.48 ± 0.47^a^	6.33 ± 0.66^b^
Total ash (%)	12.87 ± 0.1^a^	16.50 ± 0.01^b^	17.61 ± 0.23^c^	17.52 ± 0.02^c^
Crude protein (%)	20.69 ± 0.27^d^	19.04 ± 0.05^b^	17.84 ± 0.06^a^	19.64 ± 0.05^c^
Crude fat (%)	7.34 ± 0.01^b^	9.68 ± 0.17^d^	8.56 ± 0.09^c^	3.82 ± 0.04^a^
Total soluble carbohydrate (%)	1.77 ± 0.28^a^	2 ± 0.06^a^	3.34 ± 0.41^b^	3.57 ± 0.26^b^

Note: FW: fresh weight, DW: dry weight. Each value represents the mean ± standard deviation of three dry weight (DW) determinations. The average in rows marked by different letters varies significantly (*p* < 0.05).

**Table 2:** The phenolics and ascorbic acid concentration in mulberry leaves.

Chemical compositions	*Morus nigra*	*Morus nigra* sp	*Morus rubra*	*Morus alba*
Total phenolics (mg GAE/100 g DW)	461.5 ± 0.36^d^	338.7 ± 0.99^b^	434.6 ± 2.33^c^	164.9 ± 3.96^a^
Ascorbic acid (mg/100 g of DW)	3.84 ± 0.74^b^	1.66 ± 0.01^a^	3.17 ± 0.03^b^	3.14 ± 0.18^b^

Note: Each value represents the mean ± standard deviation of three dry weight (DW) determinations. The average in rows marked by different letters varies significantly (*p*  <  0.05).

**Table 3:** Correlations between different experimental parameters.

	Fat	Protein	Carbohydrate	TPC	Ascorbic acid	FRAP
Fat	1					
Protein	−0.40	1				
Carbohydrate	−0.55	−0.55	1			
TPC	0.67	−0.04	−0.53	1		
Ascorbic acid	−0.48	0.40	0.12	0.32	1	
FRAP	0.87	−0.76	−0.09	0.61	−0.35	1

## Data Availability

The datasets used and/or analyzed during the current study are available from the corresponding author upon reasonable request.
